# Implementation of Stroke Prevention Intervention Make My Day in Swedish Primary Healthcare

**DOI:** 10.1177/00084174261421395

**Published:** 2026-03-05

**Authors:** Emelie Mälstam, Eric Asaba, Elisabet Åkesson, Susanne Guidetti, Ann-Helen Patomella

**Keywords:** Activities of daily living, Health behavior, Health literacy, Healthcare disparities, Interdisciplinary, Activités de la vie quotidienne, comportement en matière de santé, disparités en matière de santé, interdisciplinaire, littératie en matière de santé.

## Abstract

**Background.** Studying the implementation of occupation-based stroke prevention interventions in primary healthcare is necessary and will provide insights of interventions’ feasibility in clinical practice. **Purpose.** To explore the implementation of the Make My Day (MMD) stroke prevention intervention in Swedish primary healthcare with health professionals (HPs) and among persons at risk for stroke. **Method.** The Medical Research Councils process evaluation framework domains, implementation, mechanisms of impact and context, guided investigation of the implementation. Data were collected through logbooks, fieldnotes, and interviews. Descriptive statistics were used to analyze process data while thematic analysis was applied to explore experiences of and interactions with the MMD intervention and contextual influences. **Results.** There was a high belief in using engaging occupations to facilitate lifestyle habit change among HP's and persons at risk for stroke. The HP's complementary competencies, sharing experiences within groups and digital self-monitoring was valued. Time constraints and age differences in groups impacted MMD delivery. Reaching a representative sample, and different Swedish primary healthcare settings was challenging. **Conclusion.** The occupation-based framework and intervention implementation was feasible yet sustainability in Swedish primary healthcare requires securing HP staff and time to deliver prevention like MMD, and reaching hard-to-reach groups in the study population.

## Résumé

**Description.** L'étude de la mise en œuvre d'interventions de prévention des accidents vasculaires cérébraux (AVC) fondées sur l'occupation dans les soins de santé primaires est nécessaire et permettra de mieux comprendre leur faisabilité dans la pratique clinique. **But.** Explorer la mise en œuvre de l'intervention de prévention des AVC Make My Day (MMD) dans les soins de santé primaires suédois auprès de prestataires de soins de santé et de personnes présentant un risque d'AVC. **Méthodologie.** Les trois domaines du cadre du Medical Research Council pour l'évaluation de processus (mise en œuvre, mécanismes d'impact, contexte) ont guidé l'exploration de la mise en œuvre. Les données ont été recueillies au moyen de journaux de bord, de notes de terrain et d'entrevues. Des statistiques descriptives ont été utilisées pour analyser les données, tandis qu'une analyse thématique a été appliquée pour explorer les expériences et les interactions avec l'intervention MMD ainsi que les influences contextuelles. **Résultats.** Les prestataires de soins de santé et les personnes présentant un risque d'AVC ayant participé à l'étude croyaient fortement que le recours à des occupations intéressantes pouvait faciliter le changement dans les habitudes de vie. Les compétences complémentaires des prestataires, le partage d'expériences au sein des groupes et l'autosurveillance numérique étaient valorisés. Les contraintes de temps et les différences d'âge dans les groupes ont eu un impact sur la prestation de l'intervention MMD. Il fut difficile d'obtenir un échantillon représentatif et de joindre différents milieux de soins de santé primaires en Suède. **Conclusion.** Le cadre fondé sur l'occupation et la mise en œuvre de l'intervention étaient faisables, mais pour en assurer la durabilité dans les soins de santé primaires suédois, il faudrait assurer la disponibilité de prestataires de soins de santé, offrir assez de temps pour réaliser une telle intervention préventive, et arriver à joindre les groupes difficiles à atteindre au sein de la population étudiée.

## Introduction

Stroke remains a major cause of morbidity and mortality worldwide (GBD, 2024; Zhang et al., 2025), yet evidence shows that up to 90% of strokes may be preventable through lifestyle changes targeting modifiable risk factors (Bushnell et al., 2024). In Sweden, the National Board of Health and Welfare (2024a, 2024b) recommends that health professionals (HPs), including Occupational Therapists (OTs), contribute to primary stroke prevention by brief structured lifestyle advice. Such advice alone, however, often fails to result in sustainable behavioral change (Albarqouni et al., 2022).

As lifestyle risk factors frequently clustered together (Stanaway et al., 2018) multifactorial interventions that promote overall healthy lifestyles have been recommended (Hall et al., 2024; Sisti et al., 2018). Yet, in Swedish primary healthcare there is a scarcity of evaluated tools that can support overall lifestyle change. HP's also face barriers in work with prevention, limited knowledge about prevention, including insufficient guidelines, constrained resources, and organizational challenges, such as inadequate leadership and support structures (Nilsing Strid et al., 2024; Patomella et al., 2018; Wood et al., 2013).

OTs are, however, a well-positioned profession to address lifestyle-related risks due to their expertise in understanding how habits and life contexts shape, and are shaped by, health and participation in occupation (Fritz & Cutchin, 2017). In other countries than Sweden, OT's work with occupation-based lifestyle interventions that have been developed for diverse populations, emphasizing empowerment, patient-centeredness, and contextualized support (Hirvonen & Johansson, 2023; Pyatak et al., 2022; Lévesque et al., 2019; Ng et al., 2013). This suggests a promising avenue for advancing stroke prevention in primary healthcare settings in Sweden.

As a response, the Make My Day (MMD) intervention was developed for Swedish primary healthcare as a novel, multifactorial, occupation-based approach to primary stroke prevention (Patomella et al., 2019). Drawing on decades of Swedish research in occupation-based stroke rehabilitation (Bertilsson et al., 2014; Guidetti et al., 2022; Gustavsson et al., 2018), MMD combines lifestyle analysis, group-based sessions, and digital self-monitoring. It targets persons at risk for stroke—to prevent a stroke to occur—by supporting them in addressing multiple lifestyle-related risk factors through engagement in everyday activities. Central to MMD is a nonpatronizing, person-centered approach that emphasizes daily routines, engaging occupations (EO's), and self-management of health and lifestyle (Patomella et al., 2019).

A central feature of MMD is the integration of EOs—activities that are meaningful, purposeful, and often experienced as motivating and restorative (Jonsson, 2008; Mälstam et al., 2022). EOs may span social, physical, creative, or household domains. While not all EOs directly benefit physical health—and some may even be detrimental when performed excessively or combined with risk behaviors (Asaba et al., 2022; Gätz et al., 2023; Kiepek et al., 2019; Widmark & Fristedt, 2019)—their potential lies in their regular integration into everyday life and their strong motivational qualities (Jonsson, 2008; Mälstam et al., 2022). EOs have not been studied previously as integrated in stroke prevention, reports on EOs have however shown increased motivation for engagement and participation in activities that hold value both for the individual and others (e.g., family, friends, or society at large) (Jonsson, 2008; Mälstam et al., 2022; Widmark & Fristedt, 2019). Previous research also shows that participation in valued occupations can increase adherence to lifestyle changes and enhance health literacy (Mälstam et al., 2022). By leveraging EO's, group support, and mobile health technology (mHealth), MMD aims to foster sustainable lifestyle changes that extend beyond traditional health advice. This makes MMD a timely and much-needed contribution to stroke prevention in Swedish primary healthcare (Mälstam, 2023).

While novel interventions such as MMD have shown promise (Mälstam et al., 2023a, 2023b; Patomella et al., 2021), their feasibility also depends on what facilitates or hinders their implementation in the healthcare organization and among the target population. In Swedish primary healthcare, implementation challenges have, for example, been lack of time and resources to implement new approaches and unclear routines for practice (Brycke, Bråndal & Brogård, 2024). Decision-makers also emphasize that incentives, resources, and organizational readiness are crucial conditions for successful implementation of digital and preventive interventions (Brantnell et al., 2020). An international example shows that health promotion interventions supported by mHealth, among older adults, need to be perceived as useful and beneficial among participants, and that a lack of availability support and resources can be challenges in the implementation (Turcotte et al., 2024). Identifying the usefulness, benefits, as well as organizational readiness of MMD in Swedish primary healthcare is therefore crucial for understanding the conditions under which MMD can be successfully implemented and sustained in practice.

The MMD pilot trial (Mälstam et al., 2023a) that preceded this evaluation demonstrated that key outcomes (i.e., stroke risk reduction) was sensitive to change, recruitment was timely and that there was an overall high response rate and study completion. Building on these findings, the present study complements the pilot trial by providing a process evaluation of the intervention's implementation. Conducting such evaluations during early developmental stage of an intervention is vital for optimizing both design and evaluation strategies for full scale trials (Moore et al., 2015).

The aim of this study was to explore the implementation process of the stroke prevention intervention MMD in Swedish primary healthcare settings (i.e., primary healthcare centers and primary healthcare rehabilitation clinics) in two different regions in mid-east Sweden to gain further knowledge for the continued development of the intervention program.

Research questions:

RQ1: Who was reached by the MMD intervention in terms of (a) primary healthcare settings (i.e., type of unit in primary healthcare, and geographical location), and (b) persons at risk for stroke?
RQ2: What in the MMD intervention was delivered and how was the delivery achieved?
RQ3: How did HP's and persons at risk for stroke experience the proposed mechanisms of impact?
RQ4: What contextual factors influenced the implementation of the MMD intervention?

## Method

### Study Design

The study was conducted as a process evaluation (Moore et al., 2015) using the Medical Research Councils (MRC) guidelines to identify and investigate key processes in the implementation of MMD, complementing results from the parallel MMD pilot trial (Patomella et al., 2019) (Clinicaltrials.gov: NCT03730701). The three MRC framework domains implementation, mechanisms of impact, and context, were utilized exploring the following aspects: recruitment of primary healthcare settings, HPs, and persons at risk for stroke (reach), what was delivered and how this was achieved (dose and fidelity), how HP's and MMD participants experienced and engaged with the intervention and how this was perceived to promote change (mechanisms of impact), and how contextual factors impacted the implementation (context). Ethical approval was obtained from the Swedish Ethical Committee (D.nr 2015/834-31, 2016/2203-32, 2019-01444, 2020-03822). Participation in the study was based on both written and oral informed consent.

### Setting

The study was conducted in close collaboration with Swedish primary healthcare, which is delivered through public and private management, and despite management model paid for by regional and municipal taxes. Meaning in this study, that participants regardless of the form of care, private or public facility, paid the same fee as usual care. Every county council, local authority, or municipality is responsible for managing and prioritizing healthcare resources and are therefore differently organized in, for example, larger primary healthcare centers or primary healthcare rehabilitation clinics. The goal of the present study was to recruit and test MMD in both types of settings in Sweden, including large city: Stockholm metropolitan area, and rural areas in the mid-north-east Sweden.

### Study Participants

Participants in this study were twofold. First, participants were persons at risk for stroke, referred to as MMD participants. The criteria for inclusion of the MMD participants were: (a) aged 45–75 years; (b) had three or more risk factors for stroke according to a stroke risk score card; (c) had access to a smartphone or wireless device; (d) expressed motivation for change; and (e) understood the Swedish language, as the mHealth app was only available in Swedish. The criteria for exclusion were: (a) having a history of a previous stroke or transient ischemic attack (TIA), and (b) expressed ongoing abuse of alcohol or drugs.

Second, participants were OTs, Physiotherapists (PTs), and Dietitians (Ds) from recruited primary healthcare sites, referred to as the HPs, and two interventionist researchers co-delivering the intervention. Inclusion criteria for primary healthcare sites were having registered HPs, at least one of each profession.

### Recruitment

Over the period of August 2018–November 2019, recruitment of primary healthcare sites was conducted. Two rural settings (primary healthcare centers) and three settings in the Stockholm metropolitan area (primary healthcare rehabilitation clinics), two of which were privately operated, agreed to participate. The sites managers were to facilitate the provision of HP's to deliver MMD. Interventionists researchers, both OTs by profession and developers of the MMD framework, were responsible for coleading MMD with the HP's.

The research team, including four OTs (one male and three female) and a physician (female), was responsible for recruiting MMD participants through local newspapers, the university social media platform, physical flyers, and digital posters. Enrollment of MMD participants to participate in the intervention was scheduled between June 2019 and June 2020, and randomization to either intervention group (IG) or control group (CG) followed after MMD participants had given their consent to participate in the study.

### The Make My Day Intervention Program

**Figure 1.**
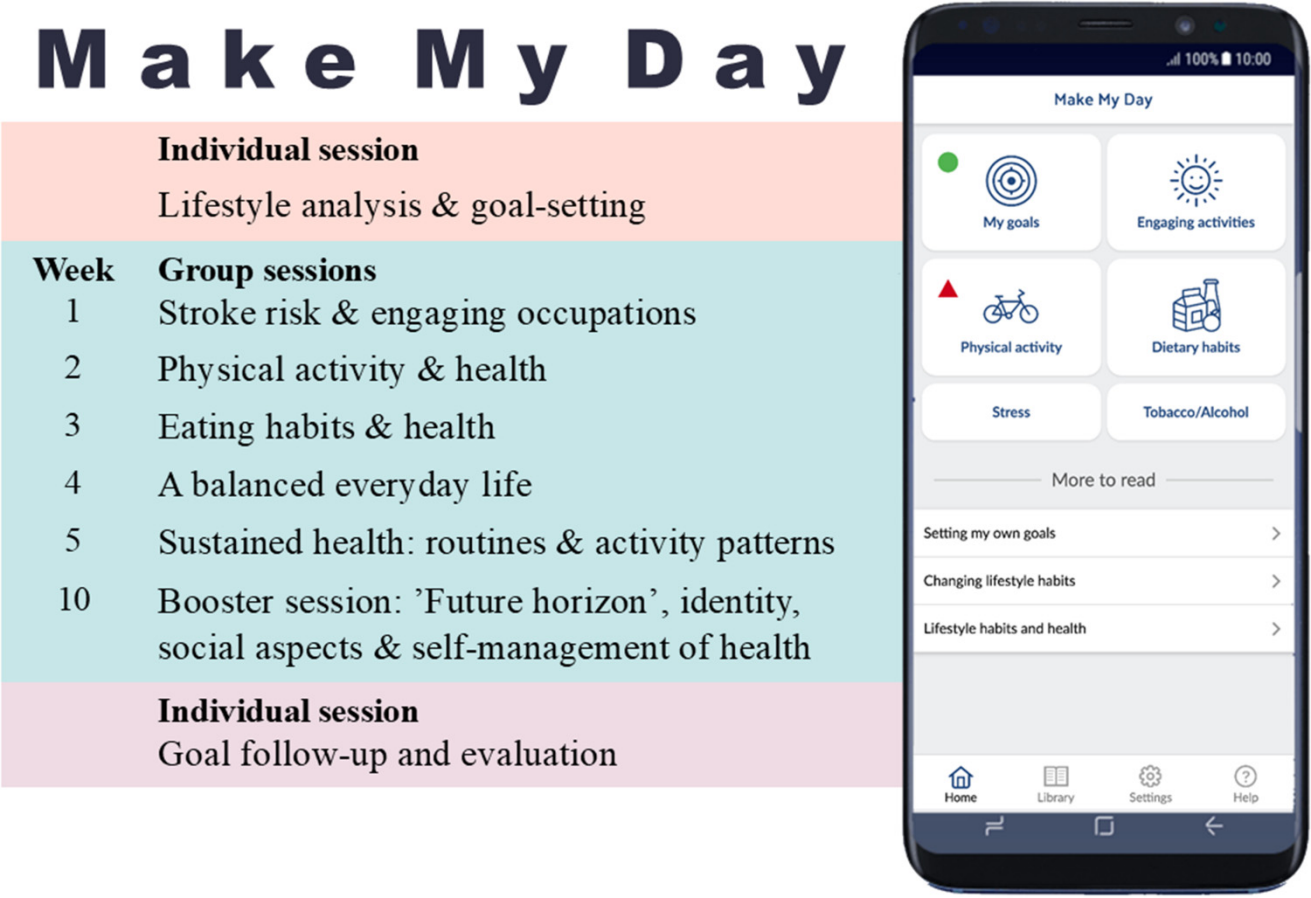
Make my day intervention content and outline.

The MMD intervention program (Patomella et al., 2019) included participation in an individual lifestyle analysis, a 10-week occupation-based intervention with six group-sessions, participation in different health promoting activities (e.g., physical exercise, food-lab, group-discussions), and individual digital self-monitoring (Figure 1).

An individual lifestyle analysis was initially performed by an interventionist researcher (OT as profession) to commence participation in MMD. It included an overview and analysis of the individuals’ whole lifestyle, including lifestyle habits (i.e., physical activity habits, eating habits, alcohol, and tobacco habits), key stroke risk factors such as blood pressure and atrial fibrillation, perceived psychosocial stress, and occupational balance.

Through motivational interview techniques, participants formed personally relevant lifestyle goals using the Canadian Occupational Performance Measure (Law, Babtiste & Mills, 1991). Lifestyle goals were integrated into the MMD mHealth app, and digital self-monitoring of goals, habits, and EOs was used to augment the group-based program. The CG underwent the same lifestyle analysis, including individual goal setting, and follow-ups as the IG, but did not receive group meetings with intervention activities and the digital tools with self-monitoring.

To study the implementation of MMD, a logic model was outlined and used to describe the needed and available resources, activities, outputs and potential outcomes, and impacts of the intervention (see Supplementary Material A).

### Implementation of MMD Intervention: Training HPs and Research Process

**Figure 2.**
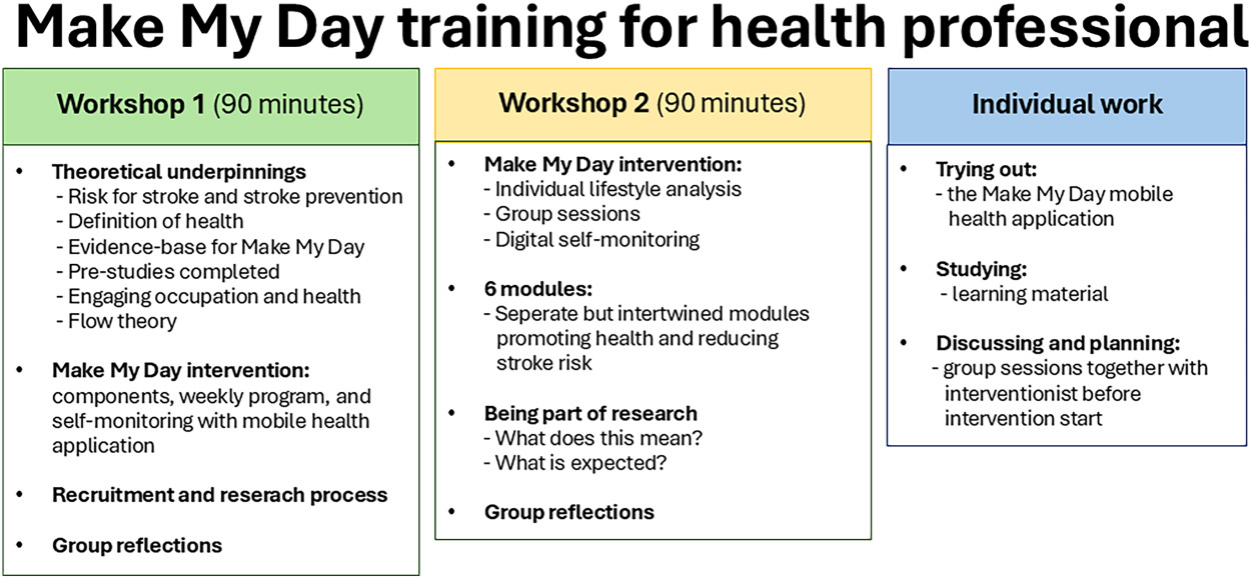
Overview of the educational content and training of health professionals.

HPs were required to participate in two educational workshops before the intervention started (Figure 2) with the aim of deepening their knowledge of the theoretical foundation of the MMD intervention (Patomella et al., 2019).

The interdisciplinary team then led MMD together with the interventionist researchers. Two researchers and a research assistant (all registered OTs) were responsible for performing the individual lifestyle analysis in MMD, the baseline and follow-up assessments in both the IG and CG reported in the pilot trial (Mälstam et al., 2023a). See Supplementary Material B for an overview of the responsible parties for each step in the research process.

### Data Collection

**Figure 3.**
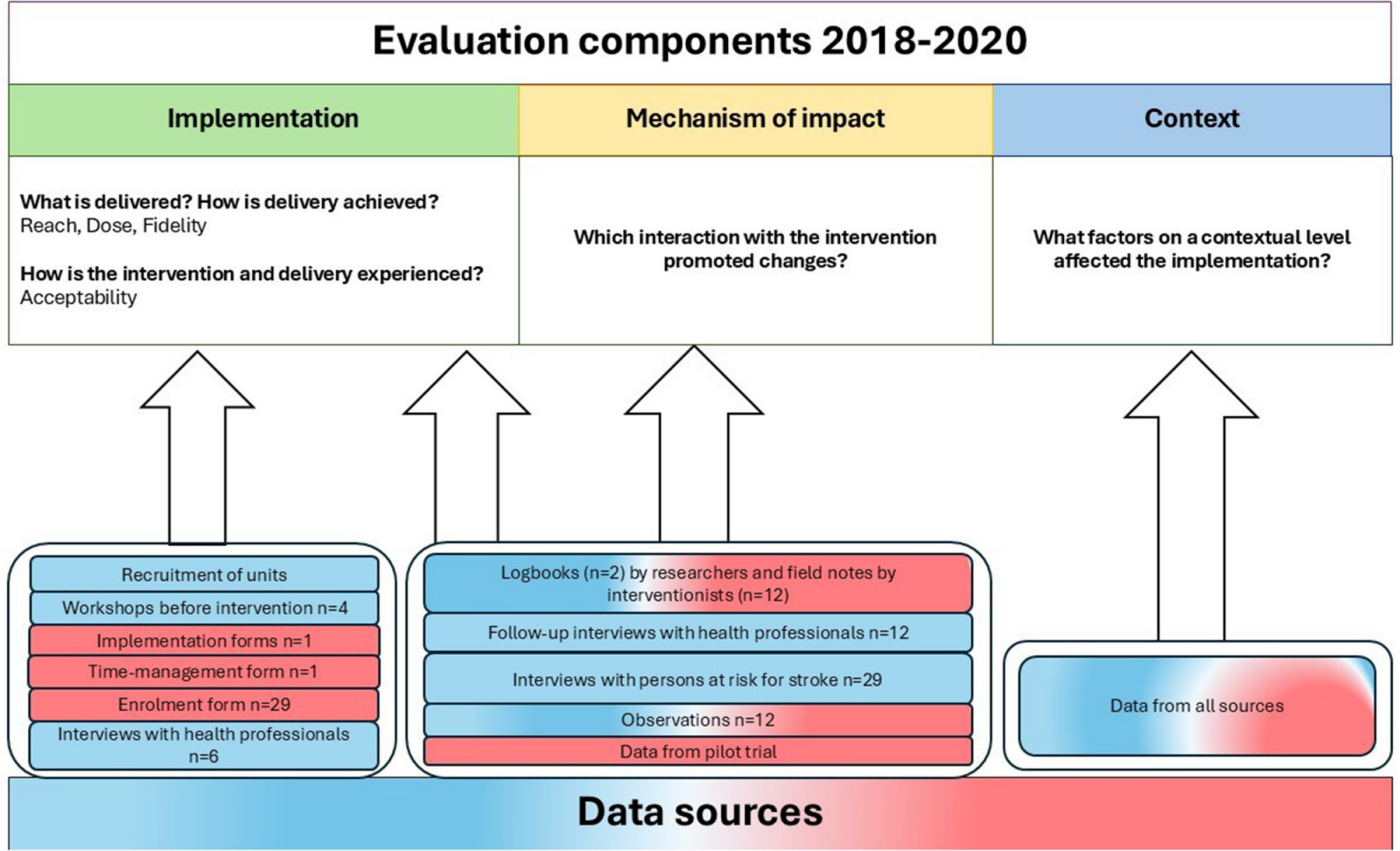
Overview of the evaluation components, research questions, and data sources. *Qualitative data sources are in red and quantitative sources are in blue.

Data collection was carried out between August 2018 and November 2020 in parallel with the MMD pilot trial (Mälstam et al., 2023a; Figure3). The intervention and group-sessions in MMD was implemented between August 2019 and February 2020. In line with the MRC framework (Moore et al., 2015), different quantitative and qualitative data were collected to evaluate the implementation, context, and mechanisms of impact of MMD. The modes of data collection were selected to get as comprehensive data as possible, combining different type of data collection, at several time points, developed based on previous research about MMD (Patomella et al., 2021).

All involved researchers followed a preformulated data collection manual, including information of how to use different instruments and document the research process. Logbooks had been used in previous stroke rehabilitation intervention studies (Guidetti et al., 2020; Kamwesiga et al., 2018) and were adapted and used to the present study documenting the study procedures weekly, including information on time of recruitment, reach, content, and extent of the educational workshops with HPs, and relevant outcome data. Field notes were written by the interventionists directly following the sessions and by research assistants during data collection, multiple information was documented: dose and fidelity of the intervention activities, follow-up reflections with the HPs and participants, and participant interactions and responses to the intervention, and contextual influences that may have impacted the implementation of the intervention.

**Figure 4.**
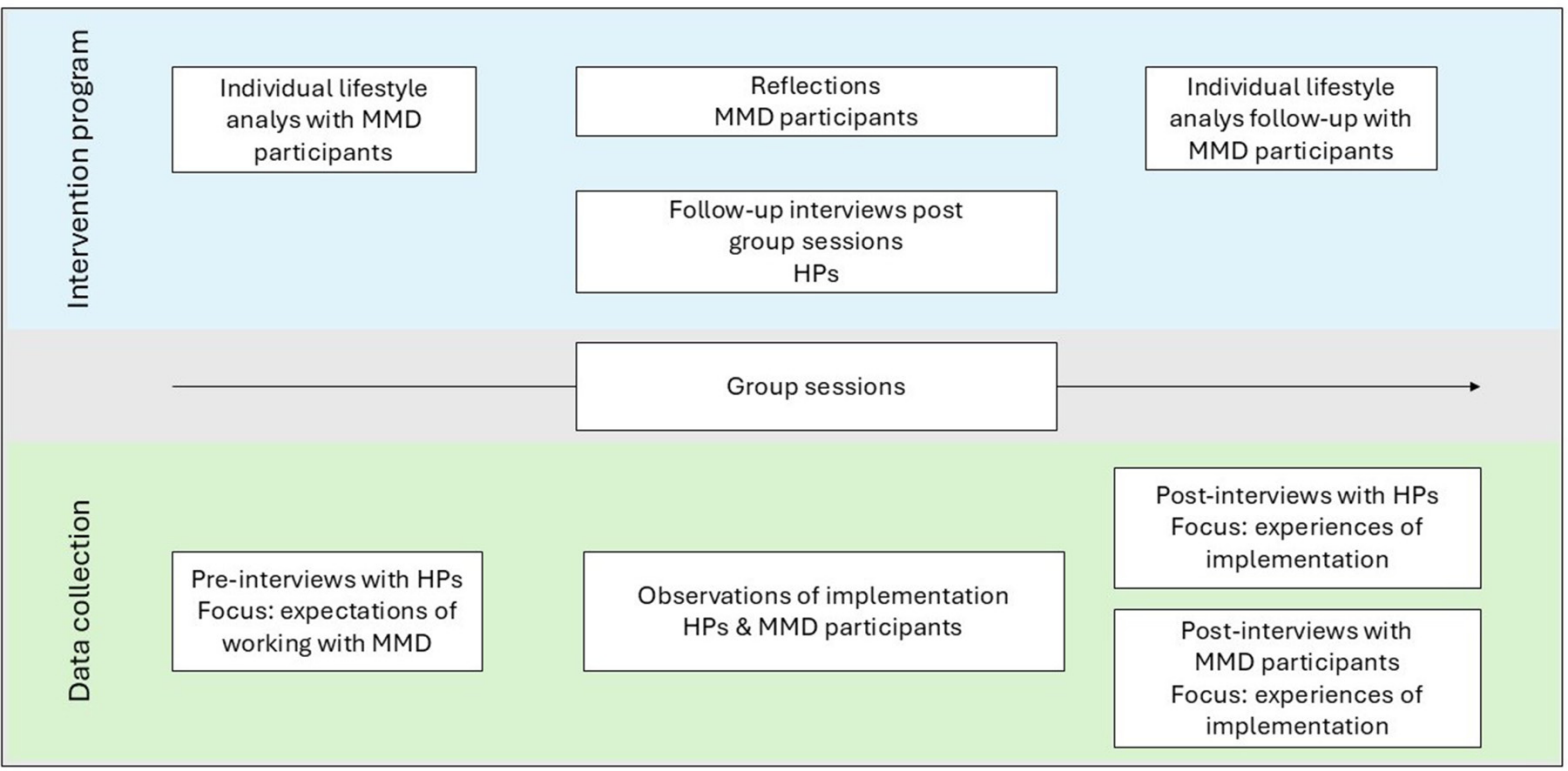
Overview of the timeline and focus of interviews.

Semidirected interviews, with HPs and persons at risk for stroke, also documented participant interaction with the intervention, context, and the experiences of MMD and its implementation. Interviews were conducted at several time points: before baseline (with HPs), during the intervention (HPs and MMD participants), and after the intervention ended (HPs and MMD participants; Figure 4). Interview guides for these data collections’ were developed within the research group based on previous studies about MMD (Asaba et al. 2022; Mälstam et al. 2022; Patomella et al. 2018; Patomella et al. 2021) and piloted within the research group and colleagues with clinical affiliation, to test comprehension of questions.

### Data Analysis

Reach, dose, and fidelity were analyzed by comparing planned procedures from the pilot study protocol (Patomella et al., 2019) with what actually happened as the study was implemented. Process data and sociodemographic data were analyzed using descriptive statistics, displayed with counts, percentages, median, and interquartile range (IQR). Dose received of MMD activities and group-sessions were analyzed and presented with median and range, the MMD app user engagement was analyzed by determining the percentage of days in the 10-week intervention period that the participant effectively used the app, presented with percentage and SD. Analysis of usability and the mHealth application's acceptability among MMD participants have been explored and reported in a separate manuscript (Mälstam, 2023b).

HPs and MMD participant’s interactions with, and experiences of, the intervention, and contextual factors were analyzed by using a thematic analysis (Braun & Clarke, 2006). All text material was used, including verbatim transcribed interviews with HPs and MMD participants and analyzed through six steps. The researchers, with both clinical and research background in Occupational Therapy and Medicine, actively engaged with the data. The first author (E. Mälstam) started by reading all text material, followed by inductively attaching codenames to chunks of text. Regular member reflections were performed together with co-authors (one male and one female) to reflect on and develop the codes. Initial themes were formed by grouping similar codes together, also discussed among coauthors. As a second step themes were situated within a deductive logic of the MRC framework domains: implementation, mechanisms of impact, and context (Moore et al., 2015), shaping the final presentation of the result. Quotes from HP interview data and excerpts from the fieldnotes are presented to illustrate the generated findings.

For qualitative data analysis, ATLAS.ti was utilized, and for quantitative analysis: the Statistical Package for the Social Sciences 22 package.

### Amendments to the Study Procedures

This study was conducted during the Coronavirus Disease 2019 (COVID-19) pandemic, necessitating methodological modifications. Amendments are described following the CONSERVE (Orkin et al., 2021) reporting guidelines. The MMD intervention program was not affected by restrictions, however, follow-up assessments were to take place in primary healthcare settings (Patomella et al., 2019) and due to COVID-19 pandemic resulting in societal restrictions (after March 2020) study procedures had to be assessed through a risk and consequence analysis. As a response to adhere to restrictions, we adapted to remote data collection by setting up a “mobile lab” and meeting the participants outside their homes.

## Results

The results are presented according to the MRC process evaluation domains. Under Implementation, we report aspects of reach, dose, and fidelity. Under Mechanism of impact, HPs and MMD participants’ interactions with and experience of the intervention is described. Under context, we describe contextual factors that influenced the interventions implementation.

### Implementation

#### Reach

Out of 83 individuals expressing interest *n* *=* 34 (41%) met the inclusion criteria to be randomized to the IG or CG, with *n* *=* *5* (10%) drop-out (Supplementary material C). Modes for recruitment included local newspapers (*n* = 11), social media (*n* = 15), and personal referrals by friends or family (*n* = 3).

Study participants ages were 49–73, and most were women (*n* = 20), Swedish-born (*n* = 20), and living in neighborhoods with moderate to high socioeconomic conditions (*n* = 24). The main barrier to eligibility was reported as difficulties to participate in an intense onsite intervention (10 weeks) during working hours during (*n* = 21). Table 1 shows the main sociodemographic factors among the IG.

**Table 1:** Study Population Demographics

Variables	IG *n*=14
Age in years, *mean (SD)*	61.9 (8.5)
Education years, *mean (SD)*	13.5 (1.8)
Sex, female (%)	8 (57)
Country of birth, Sweden (%)	9 (64)
Living situation, *count* Single household (%)	8 (57)
Area of living//neighborhood,^a^ *count* Moderate to high socioeconomic condition	11 (79)
Work status count Working	6
Not working^b^	8
Yearly income, in euro, *count* >58,000	3
<58,000 >19,300	10
<19,300	1

^a^ According to the Swedish National Board of Housing, Building and Planning (https://segregationsbarometern.boverket.se/labbet/#/omradesstatistik/map?indicator=0-1,2,3,4,5&location=riket&bg=0).

^b^ Persons that were not working were either retired (*n* = 9), unemployed (*n* = 2), or on sick leave (*n* = 3).

Due to a shortage of OTs in rural primary healthcare sites, because of high clinical demands and staffing goals that could not be met, MMD was only delivered at two of the five planned sites, and therefore with less participants in the IG (*n* = 15) than planned for in the study protocol (*n* = 30). Included sites were located in the metropolitan area of Stockholm, Sweden, one in public regime and the other in a private regime. The two HP teams (*n* = 6) delivering the MMD intervention had a similar composition regarding age, median = 29.5 (IQR 5) and work experience in years, median = 2.5 (IQR 2).

#### Dose and Fidelity

Training of HPs (*n* = 6) included two 90-min workshops. Each group of HPs were introduced to the MMD theoretical underpinnings and intervention, recruitment and research process, and reflected and discussed about their work, the MMD, and the key concepts in the framework, such as using EOs, digital self-monitoring, and goal-setting. Each team implemented six MMD group sessions each, including group activities, discussions about EOs together with the MMD participants, and following up the digital self-monitoring of lifestyle goals. At both sites, MMD participants were involved in choosing what kind of activities to perform each week. Differences in the two primary healthcare unit's physical premises were described in the fieldnotes to impact on the physical activities that were decided on, and performed. See Supplementary Material D.

The HPs also described time constraints, at follow-up interviews, to fit everything into each group session, while maintaining fidelity to the program framework: “Everything works, but it is difficult with time. 90 min is not enough” [Interventionist 2 fieldnote (I2F)]. Limitations in time were described to lead to different topics overlapping during the weekly sessions. While session overlapping was not inherently problematic, as themes were described to naturally share content, HPs expressed having difficulties in fitting all content into the six group-sessions with only 90 min for each session. HPs also explained that as some participants were in retirement age and had more time to work with lifestyle goals and EO's, whereas participants in working age had a busier everyday life and a harder time to fit changing lifestyle habits and EOs into their schedule. The age difference in the groups was described at several times to be a barrier to support the participants.

Regarding dose of the intervention, the number of attended IG sessions was lower than planned in the protocol at both sites *n* *=* 4.5 out of 6. The app user engagement was high at a group level, with a mean number of valid days across the 10-week intervention period of 71.4% (SD = 10.7). Participants used the MMD app on average 4.7 out of 7 days (63.0% per week, SD = 0.6). See Table 2 for detailed data on the dose received in each site.

**Table 2:** Activity and Dose Received from the MMD Intervention for the MMD Participants in Terms of Group Sessions and mHealth App Engagement

Activity and dose according to protocol	Dose according to protocol	Dose received by MMD participants
Group sessions		
*Number of sessions attended Site 1, median (range)*	6 (4–6)	6 (2–6)
*Number of sessions attended Site 2, median (range)*	6 (4–6)	4 (3–6)
*Number of sessions attended Site 1+2, median (range)*	6 (4–6)	5 (2–6)
App usage		
Use of the MMD mHealth app, *mean (SD)*	7 days a week for 10 weeks	4.7 (0.57)

#### Mechanisms of Impact

The analysis of mechanisms of impact revealed three components that impacted the HPs and participants interaction with the intervention. Among these, the use of EOs emerged as the most prevalent and influential mechanism, consistently described by both HP's and participants as highly valued for promoting lifestyle change. While group-based learning and interdisciplinary collaboration were also central, their perceived impact was more context-dependent and occasionally constrained by time and group composition. Taken together, all three mechanisms contributed to lifestyle habit change, yet EOs stood out as a unifying thread—integrated into and reinforcing the other components, making them and the focus of overall health, a cornerstone of meaningful and sustainable lifestyle change.

##### Using EOs as a Tool for Prevention

Features highly valued in MMD were the focus on overall health, incorporating healthy activity patterns in everyday life, and using EOs as tools for prevention. Building on EOs to promote healthy habits and incorporating the experiential components (e.g., food lab, Tabata exercise, yoga, walk‘n’talk) in group-setting was a new way of working for the HPs, but, both HPs and MMD participants enjoyed it: “It was easy for the participants to relate to the concept after the HP's explanation and participants reported having had EOs previously, but not everyone had EOs today. Some also wanted to find new EOs and contexts for this, as current EOs were not promoting their health, or were not an option anymore” [I2F]. Participants also had different possibilities of participation in EOs. Due to factors such as employment status, financial or social resources, and living conditions. HPs described that even if they like the approach, the focus of overall lifestyle (several risk factors) could, at the same time, be challenging: “Overall, I like the approach, but I also think that it can become too much. Patients may feel … it can be too many focuses and might be too much to deal with at once” [HP1]. To continue working with MMD this would require the HPs having enough time and knowledge about the situatedness of EOs and lifestyle habit change, to support participants.

##### Sharing Experiences and Learning Together

The group format and learning together in the group were described as important aspects of MMD facilitating change. This was both the way the HP's wanted to work and thought to be suitable for the purpose of lifestyle habit change: “The method with the target group is positive, to work in groups and for participants to meet others in groups” [HP2]. The group provided a safe space for increasing awareness about stroke risks and health, which seemed to create a sense of belonging among the MMD participants that was important in the process of changing lifestyle habits:

“It was not just me cheering them on, encouraging them. They did this together. Also, they felt that they were not the only one that had to work with this, that thought that it was difficult, experiencing cravings for snacks, thinking it is challenging to work out, getting tired right away, or even thinking it is boring with physical activity. It was a collective experience, which I think was very good for them” [HP3].

Engaging in activities and reflecting together created a supportive context where shared experiences became tools to incorporate healthy lifestyle habits in everyday life: “The participants had started doing more things that engaged them. Occupations that they wanted to do, such as going to the theater, getting involved in senior activities, playing billiards” [I2F]. Barriers in relation to this mechanism were, however, time-constraints. At the start of MMD, both the participants and HPs described limited time to get to know each other, as well as learn how to use the mHealth app. In addition, it was not the HPs conducting the individual lifestyle analysis, which was expressed to limit their knowledge about the MMD participants. This hindered HPs supporting the participants during the group-sessions.

Another barrier to sharing experiences was the large age differences in the groups, which affect what daily engagements in activities the MMD participants had and wanted to discuss. This impacted both participation in groups, and delivery of the content of the intervention. One of the HPs reflected on this after a weekly MMD session: “On this occasion, the heterogeneity was evident in the group. They are all at different stages in life with different conditions” [HP1]. One of the participants was also absent from two group-sessions, leaving a notice that this was because the person did not feel a sense of belonging in the group due to age-differences.

##### Complementary Competencies Enhancing Patient Empowerment

The interactions with, and within, the team of HPs were highly valued, especially since there was a lot of complementary competence in the group as support to address the complexity of changing several lifestyle habits: “We all have a lot of experience that complements in a good way” [HP4]. It was also indicated that the way that MMD was designed, with interdisciplinary teams moderating the intervention and using a nonpatronizing approach led to a redistribution of power between the HP's and MMD participants. HPs expressed this shift positively in terms of empowerment for the participants health: “It was an engaged group, open, discussing. As they shared between themselves, I became more of a moderator. A little like that, developing it further [HP3]. They (participants) have received support and information, but the insights have come from themselves, and they have done the work themselves. A lot has happened in the group [HP1]. It is obvious that they (participants) have the power, they have the app. It may sounds strange, but it reevaluates how I look at my role. I am still a mediator of knowledge or whatever you want to call it. But maybe that the distribution of power will be a little different” [HP5].

As the MMD participants did most of the work themselves, the role of the HP's was described to shift from being the ones in charge of the program to becoming one among the group, facilitating activities and discussions. Different HPs, however, had different amount of work experience in their profession and with working with group format and lifestyle habit change, which impacted on how easy they perceived facilitation.

##### Context

Finally, two major contextual factors were found to influence the implementation of MMD, and the interaction the MMD participant's and HP's had with the intervention. First, differences in the physical premises at the two sites impacted the possibility of choosing EOs to perform in the interventions. At the private regime there were large open spaces, allowing participants to choose from various types of physical activities, while the site in the public regime had small premises, allowing only circuit gym training. At the public site, the participants had the opportunity to access a nearby green area, where they chose to perform a walk-and-talk activity, which was not possible at the other site. This contextual influence was described to impact the delivery of the intervention and possibility to keep fidelity to MMD framework of using EOs as not all participants expressed the chosen physical activities to be engaging.

Secondly, there was a discrepancy between what the HP's wanted to do, what they perceived their competencies to be, and what they were currently assigned to do in terms of prevention in the healthcare organization. Apart from their involvement in delivering MMD, they primarily focused on secondary prevention for individuals with heart failure or type 2 diabetes, even though the HP's considered themselves both willing and knowledgeable in primary prevention. One HP said: “I think there is a lot you can do by changing lifestyles through what we work with … Much more and much earlier than we do. Now it's like you have to be sick” [HP3]. The HP's competencies were described as underutilized in the healthcare organization due to the current reimbursement system in Swedish primary healthcare. The fact that the reimbursement system did not reimburse addressing modifiable risk factors for stroke through primary prevention equivalent to the work in MMD was considered as a barrier for working with MMD in primary healthcare: “If there is money to do it, if the management thinks we should go for it, we have an overflow of patients that's wants it. It depends on what we should do” [HP4]. A concern regarding the reimbursement system and the feasibility of the intervention was also expressed: “There is six sessions, but are this enough for sustainable change in someone's life? It's always a question and a pitfall” [HP6].

## Discussion

This study explored the implementation of the MMD intervention in two Swedish primary healthcare rehabilitation clinics, contributing knowledge for future development of occupation-based lifestyle interventions in this context. The result are consistent with earlier studies (Mälstam et al., 2023a; Patomella et al., 2021), suggesting that MMD can be a valuable tool in primary stroke prevention when delivered by interdisciplinary teams including OT's.

Key facilitators included strong belief in the occupation-based framework, high willingness among HP's and participants to engage in healthy lifestyle promotion, and complementary competencies in the interdisciplinary teams together with the knowledge among the participants. The finding that HPs found the use of EOs both novel and valuable in their approach to support healthy lifestyle promotion is important. Incorporating healthy activity patterns in everyday life through EOs provides support for an important role and conceptual expertise of Occupational Therapy in the area of prevention, earlier described in relation to OT's work with habit formation (Fritz & Cutchin, 2017). MMD also provides a systematic way of integrating prevention in primary healthcare settings, which has been highlighted as important for implementation success (Nilsing Strid et al., 2024; Patomella et al., 2018; Wood et al., 2013). The combination of digital components and group-based experience sharing also enhanced a sense of belonging, which may have mediated outcomes of stroke risk reduction as seen in the pilot trial (Mälstam et al., 2023a). These results all support a full-scale trial, incorporating knowledge gained in the current study and previous studies about MMD (Mälstam et al., 2023a; Patomella et al., 2021).

The result moreover indicates that even though intervention contexts were comparable and participation in and reflection on EOs were described as positive, MMD participants opportunities to participate in EOs and lifestyle habit change varied due to differences in participants’ life situations, particularly related to age, which have been seen before (Jonsson, 2011; Patomella et al., 2021). Participants, often retired, had more time and flexibility to work with lifestyle goals, while those of working age faced competing demands and time constraints that made it harder to implement changes. Other studies have shown that intergenerational groups can promote social and mental well-being and active aging (Salvino et al., 2025). Yet, the differences seen in our study influenced the relevance of group discussions and chosen activities, as interests and priorities varied across life stages, creating challenges for HPs in meeting all participants’ needs. Creating more homogeneous groups in terms of age may therefore improve group cohesion and the applicability of shared experiences. However, achieving such homogeneity is complex in primary healthcare settings, where recruitment pools are limited and interventions must remain inclusive. Future implementation in Swedish primary healthcare may need to balance these considerations by either extending session time to accommodate diverse needs or by structuring groups with narrower age ranges when feasible, without compromising accessibility and equity.

Moreover, in this study interdisciplinary teamwork and a mixed-method approach (group sessions and digital self-monitoring) were both well-received and requested, which addresses a common experience of workload in primary healthcare (Nilsing Strid et al., 2024; Patomella et al., 2018; Wood et al., 2013), and in relation to projected HP shortages. Moving forward, this approach may serve as a feasible alternative to traditional individual lifestyle counseling, which previously has been criticized (Albarqouni et al., 2022). The potential for this group of HP's (i.e., OTs, PTs, and Ds) to deliver MMD must also be underscored as the conceptual foundations of MMD aligned well with their perceived knowledge and skills. The limited amount of training the HP's participated in also worked to deliver the intervention, supporting the idea of using this constellation of HPs for MMD. However, one OT was present at each program session, securing that the occupation-based approach was implemented. A possible result connected to this may be the increased participation in EOs highlighted by the HP's and MMD participants. This points to the importance of using OT expertise to support implementation of EOs for promoting health and wellbeing in the target population.

Important results in terms of the interventions sustainability are barriers on the organizational and socio-political level. Although prevention is supposed to be high on the global and national healthcare agenda, the results of this study, in line with other studies (Nilsing Strid et al., 2024; Patomella et al., 2018), show that preventative services related to stroke risk factors are not readily reimbursed. The (un)readiness for financing novel prevention (Kabir et al., 2022) in Swedish primary healthcare was evident from the HP's descriptions of time constraints. HP's even saw a risk of not being able to keep fidelity to the theoretical framework due to this. There were also difficulties of reaching the study populations. It was not possible to reach different primary healthcare settings, particularly evident in rural swedish regions, due to a lack of available OTs. How to achieve sustainable provision of the healthcare workforce for preventative services is an ongoing issue emphasized in the European strategy for equal healthcare for all (European Patients forum, 2017), and an issue highlighted by Swedish HPs and decisions makers (Bratnell et al., 2020; Brycke, Bråndal & Brogårdh, 2024). If the shortage of OTs persists in Swedish primary healthcare, it may be relevant, especially for rural regions, to develop the mHealth support more and decrease physical sessions, however the groups were highly valued. Another option is to evaluate using other HPs to deliver MMD, yet, this might necessitate extended training in the occupation-based framework to ensure fidelity, which unfortunately could make the program less cost and time efficient.

Finally, methods used for recruitment of persons at risk for stroke may not have been sufficient to reach a broad representation of the target population. As a result, persons living in neighborhoods of low socioeconomic status, persons with an immigrant background, and those with lower education and/or income levels were insufficiently represented in the study, which is a limitation in the research and methodology used. In the continued evaluation of MMD, it is important to use relevant modes for recruitment, possibly by identifying key gatekeepers to reach “hard-to-reach” populations (Blukacz et al., 2023).

## Strength and Limitations

Strengths included that the study was conducted in parallel with a pilot trial (Mälstam et al., 2023a), allowing systematic and rigorous quantitative data collection, and iterative qualitative data collection and analysis. The development of MMD, data collection, as well as, its evaluation, was based on the MRC framework for process evaluations (Moore et al., 2015), including collecting various data sources over time, and following a data collection manual to ensure consistency across data collectors. This systematicity and methodological pluralism enabled insights into the implementation process of the MMD intervention as well as triangulation of data sources.

The qualitative result was strengthened through a reflexive approach with researchers’ active engagement with the data and critically discussing interpretations continuously within the research team. Starting the thematic analysis inductively also supported exploration of data without having predefined themes too early. While later relating the findings to the MRC domains facilitates comparisons with other process evaluations and the relevance of the results to the implementation field.

Some of the evaluations, which possibly help explain outcomes, were conducted post hoc, which can be seen as a limitation (Moore et al., 2015). For the future, presenting the results of the process evaluation first can be considered, to provide context for outcomes. Moreover, specific quality criteria were not fully developed in the pilot study protocol (Patomella et al., 2018), which limited the extent to statistically assess process outcomes. The qualitative result, however, contributes descriptive information of the process, which is a strength.

Finally, as the study was carried out in a limited number of primary healthcare settings and did not include rural areas, this reduces contextual diversity and transferability of findings. The implementation process was moreover conducted in close collaboration with the research team, which may have influenced fidelity. For future studies, broader involvement of managers, HPs, and persons at risk for stroke in co-design and evaluation processes is recommended. Such a collaborative approach could help address contextual barriers, identity support needed within specific subpopulations, and strengthen the sustainability of the intervention across diverse primary healthcare settings (Talevski et al., 2023).

## Conclusion

This study shows the feasibility and potential of an occupation-based approach for primary stroke prevention, in two Swedish primary healthcare. There was a high belief in using EOs as the main agent of change, complementary competencies in the interdisciplinary teams, and sharing experiences within groups to achieve lifestyle habit changes. Sustainable implementation of MMD, however, relies on multiple aspects: (a) securing the availability and provision of HP's, especially OTs, in Swedish primary healthcare settings, (b) changing the reimbursement system to premiere sufficient time to work with primary stroke prevention of modifiable risk factors for HPs, and (c) reaching a breadth in the at-risk for stroke population, especially hard-to-reach populations.

## Key Messages

- There was a high belief in the occupation-based intervention.
- Complementary competencies in the teams and sharing experiences within a group supported changes in lifestyle habits, but time constraints and age differences in groups were barriers for delivering the intervention.
- Sustainable implementation in Swedish primary healthcare relies on securing provision of health professionals, especially occupational therapists, and premiering their work with primary stroke prevention.

## Supplemental Material

sj-pptx-1-cjo-10.1177_00084174261421395 - Supplemental material for Implementation of Stroke Prevention Intervention Make My Day in Swedish Primary Healthcare Supplemental material, sj-pptx-1-cjo-10.1177_00084174261421395 for Implementation of Stroke Prevention Intervention Make My Day in Swedish Primary Healthcare by Emelie Mälstam, Eric Asaba, Elisabet Åkesson, Susanne Guidetti and Ann-Helen Patomella in Canadian Journal of Occupational Therapy

sj-docx-2-cjo-10.1177_00084174261421395 - Supplemental material for Implementation of Stroke Prevention Intervention Make My Day in Swedish Primary Healthcare Supplemental material, sj-docx-2-cjo-10.1177_00084174261421395 for Implementation of Stroke Prevention Intervention Make My Day in Swedish Primary Healthcare by Emelie Mälstam, Eric Asaba, Elisabet Åkesson, Susanne Guidetti and Ann-Helen Patomella in Canadian Journal of Occupational Therapy

sj-docx-3-cjo-10.1177_00084174261421395 - Supplemental material for Implementation of Stroke Prevention Intervention Make My Day in Swedish Primary Healthcare Supplemental material, sj-docx-3-cjo-10.1177_00084174261421395 for Implementation of Stroke Prevention Intervention Make My Day in Swedish Primary Healthcare by Emelie Mälstam, Eric Asaba, Elisabet Åkesson, Susanne Guidetti and Ann-Helen Patomella in Canadian Journal of Occupational Therapy

sj-docx-4-cjo-10.1177_00084174261421395 - Supplemental material for Implementation of Stroke Prevention Intervention Make My Day in Swedish Primary Healthcare Supplemental material, sj-docx-4-cjo-10.1177_00084174261421395 for Implementation of Stroke Prevention Intervention Make My Day in Swedish Primary Healthcare by Emelie Mälstam, Eric Asaba, Elisabet Åkesson, Susanne Guidetti and Ann-Helen Patomella in Canadian Journal of Occupational Therapy
