# Supplementary material for: Implementation of Stroke Prevention Intervention Make My Day in Swedish Primary Healthcare
Source: Can J Occup Ther. 2026 Mar 5;93(2):151–63. doi: 10.1177/00084174261421395 (PMC13187227; doi:10.1177/00084174261421395)
Supplement: sj-pptx-1-cjo-10.1177_00084174261421395 - Supplemental material for Implementation of Stroke Prevention Intervention Make My Day in Swedish Primary Healthcare [file sj-pptx-1-cjo-10.1177_00084174261421395.pptx]

## Slide 1
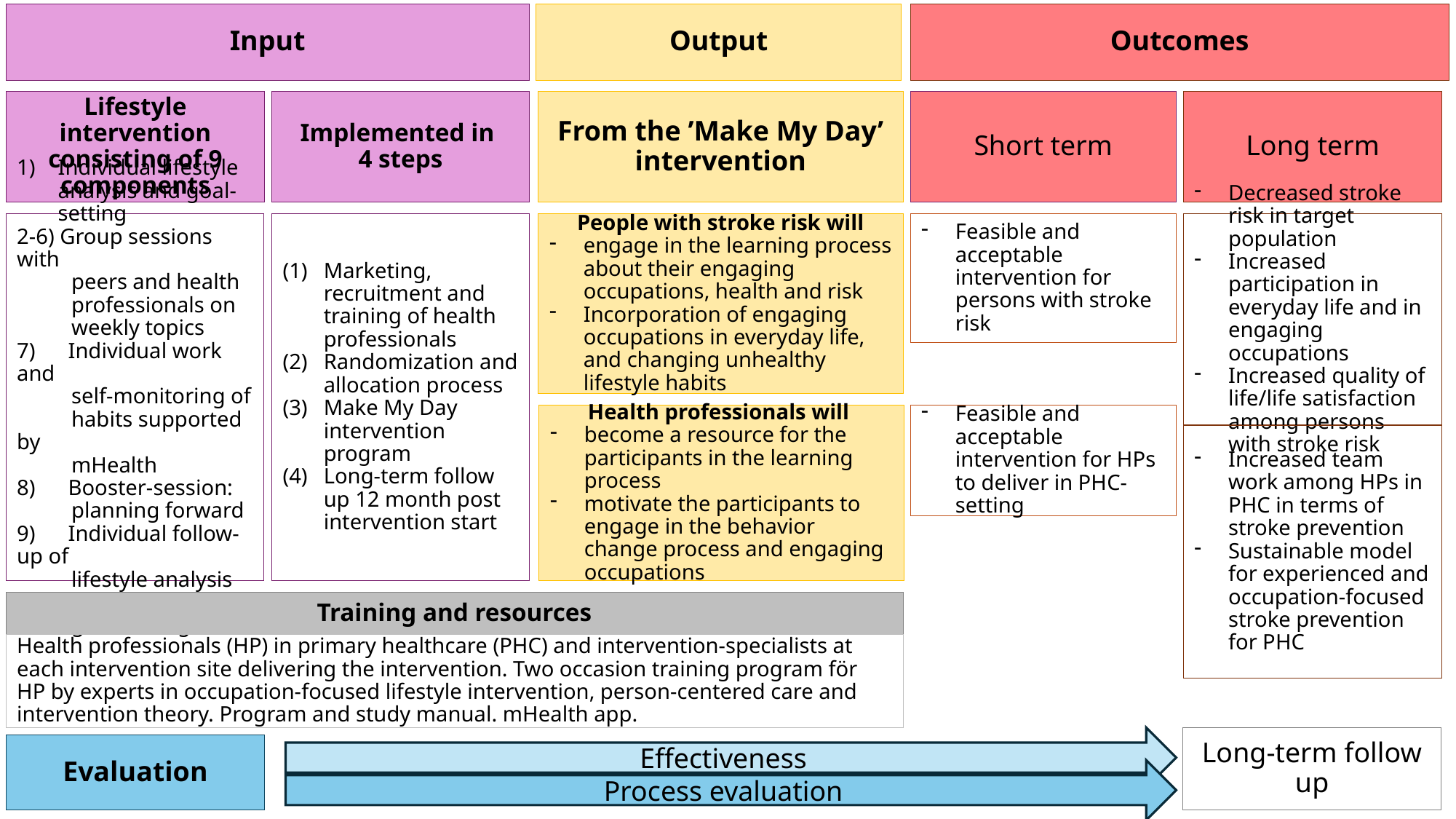

# Input
Output
Outcomes
Lifestyle intervention consisting of 9 components
Implemented in
4 steps
From the ’Make My Day’ intervention
Short term
Long term
People with stroke risk will
engage in the learning process about their engaging occupations, health and risk
Incorporation of engaging occupations in everyday life, and changing unhealthy lifestyle habits
Feasible and acceptable intervention for persons with stroke risk
Decreased stroke risk in target population
Increased participation in everyday life and in engaging occupations
Increased quality of life/life satisfaction among persons with stroke risk
Individual lifestyle analysis and goal-setting
2-6) Group sessions with
 peers and health
 professionals on
 weekly topics
7) Individual work and
 self-monitoring of
 habits supported by
 mHealth
8) Booster-session:
 planning forward
9) Individual follow-up of
 lifestyle analysis and
 goal-setting
Marketing, recruitment and training of health professionals
Randomization and allocation process
Make My Day intervention program
Long-term follow up 12 month post intervention start
Health professionals will
become a resource for the participants in the learning process
motivate the participants to engage in the behavior change process and engaging occupations
Feasible and acceptable intervention for HPs to deliver in PHC-setting
Increased team work among HPs in PHC in terms of stroke prevention
Sustainable model for experienced and occupation-focused stroke prevention for PHC
Training and resources
Health professionals (HP) in primary healthcare (PHC) and intervention-specialists at each intervention site delivering the intervention. Two occasion training program för HP by experts in occupation-focused lifestyle intervention, person-centered care and intervention theory. Program and study manual. mHealth app.
Effectiveness
Long-term follow up
Evaluation
Process evaluation
