# Supplementary material for: Implementation of Stroke Prevention Intervention Make My Day in Swedish Primary Healthcare
Source: Can J Occup Ther. 2026 Mar 5;93(2):151–63. doi: 10.1177/00084174261421395 (PMC13187227; doi:10.1177/00084174261421395)
Supplement: sj-docx-2-cjo-10.1177_00084174261421395 - Supplemental material for Implementation of Stroke Prevention Intervention Make My Day in Swedish Primary Healthcare [file sj-docx-2-cjo-10.1177_00084174261421395.docx]

**Supplementary material B.** Involved parties and research process

| **Research process** | **Site 1** | **Site 2** |
| --- | --- | --- |
| Intervention for and with persons at risk for stroke | Interventionist 1  Health professionals | Interventionist 2  Health professionals |
| Logbook | Interventionist 1 | Interventionist 2 |
| Outcome assessments | Interventionist 1  Research assistant | Interventionist 2  Research assistant |
| Field notes | Interventionist 1  Research assistant | Interventionist 2  Research assistant |
| Interviews with HP’s before MMD pilot trial | Interventionist 1 & 2 | Interventionist 1 & 2 |
| Interviews with HP’s after the MMD pilot trial | Interventionist 2 | Interventionist 2 |
