# Supplementary figures and images for: Implementation of Stroke Prevention Intervention Make My Day in Swedish Primary Healthcare
Source: Can J Occup Ther. 2026 Mar 5;93(2):151–63. doi: 10.1177/00084174261421395 (PMC13187227; doi:10.1177/00084174261421395)

Supplemantary material C. Figure of flowchart


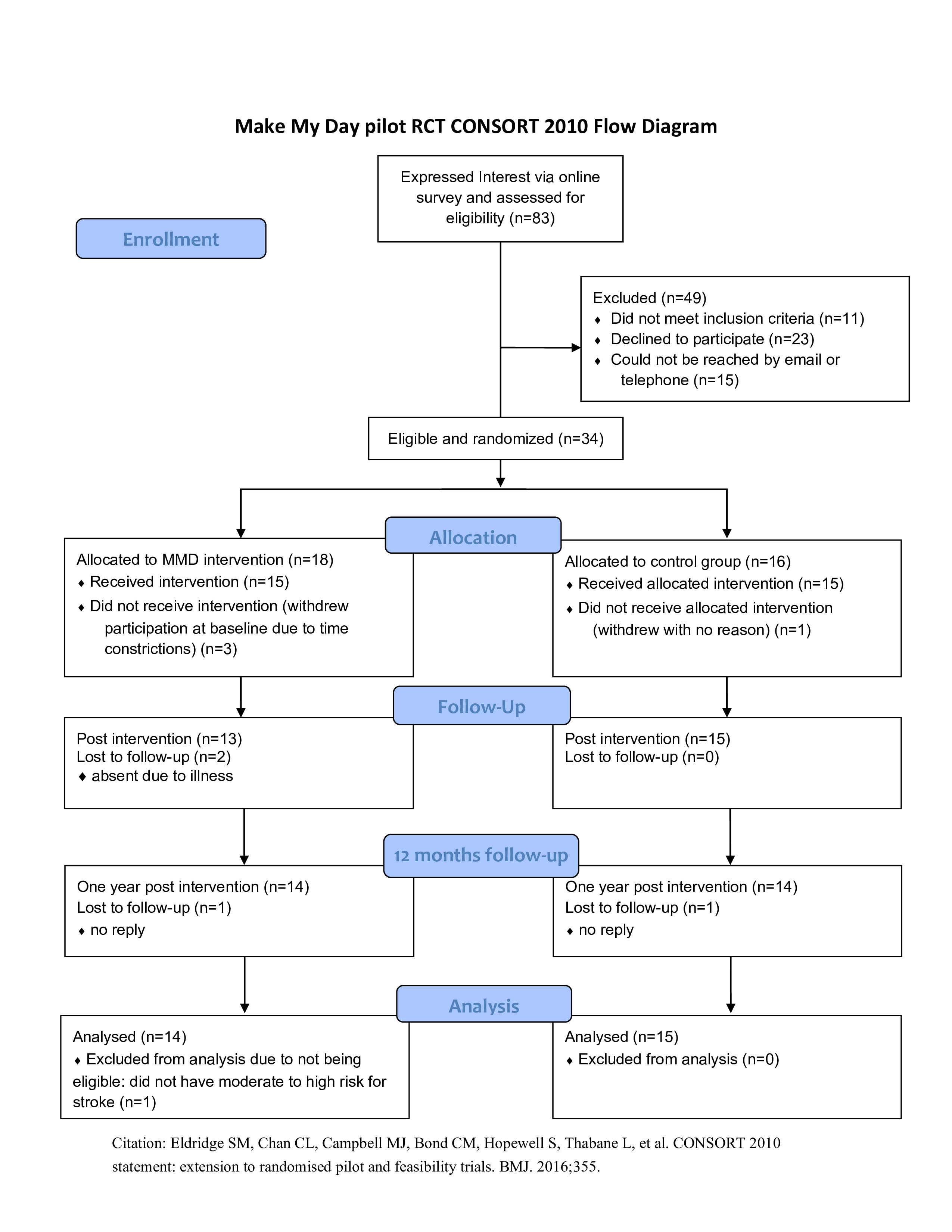

Supplement: sj-docx-3-cjo-10.1177_00084174261421395 - Supplemental material for Implementation of Stroke Prevention Intervention Make My Day in Swedish Primary Healthcare [file sj-docx-3-cjo-10.1177_00084174261421395.docx]
