# Supplementary material for: Implementation of Stroke Prevention Intervention Make My Day in Swedish Primary Healthcare
Source: Can J Occup Ther. 2026 Mar 5;93(2):151–63. doi: 10.1177/00084174261421395 (PMC13187227; doi:10.1177/00084174261421395)
Supplement: sj-docx-4-cjo-10.1177_00084174261421395 - Supplemental material for Implementation of Stroke Prevention Intervention Make My Day in Swedish Primary Healthcare [file sj-docx-4-cjo-10.1177_00084174261421395.docx]

**Supplementary material D.** Intended and performed activities in the two primary healthcare sites.

| **Intended activities** | **Site 1 - Performed activities** | **Site 2 – Performed activities** |
| --- | --- | --- |
| **Stroke risk & engaging occupations**   - Interactive learning session - Discussing own engaging occupations - Interest checklist and engaging engaging survey for self-study | - Interactive learning session - Interview pairwise - Interest checklist and engaging engaging survey for self-study | - Interactive learning session - Interview pairwise - Interest checklist and engaging engaging survey for self-study |
| **Physical activity & health**   - Interactive learning session - Trying out a chosen physical activity | - Interactive learning session - Gym-circuit-training | - Interactive learning session - Body weight-circuit-training |
| **Eating habits & health**   - Interactive learning session - Food-lab (e.g. making snack together) | - Interactive learning session - Making healthy sandwiches | - Interactive learning session - Making healthy sandwiches |
| **A balanced everyday life**   - Interactive learning session - Self-analysis of occupational balance - Relaxation exercise | - Interactive learning session - Self-analysis of occupational balance - Meditation and yoga | - Interactive learning session - Self-analysis of occupational balance - Medical yoga |
| **Sustained health: routines & activity patterns**   - Interactive learning session - Activity of choice - Group discussion | - Interactive learning session - Walk’n’talk - Group discussion | - Interactive learning session - Tabata-exercise - Group discussion |
| **Booster session: ’Future horizon’**   - Interactive learning session - Self-analysis of resources for health management - Usage of mHealth - Group reflection and Swedish “fika” (coffee and fruits) | - Interactive learning session - Self-analysis of resources for health management - Usage of mHealth - Group reflection and Swedish “fika” (coffee and fruits) | - Interactive learning session - Self-analysis of resources for health management - Usage of mHealth - Group reflection and Swedish “fika” (coffee and fruits) |

**Yellow highlighted parts shows what differed between what was planned and between the different sites .*
